# Novel homozygous BMP9 nonsense mutation causes pulmonary arterial hypertension: a case report

**DOI:** 10.1186/s12890-016-0183-7

**Published:** 2016-01-22

**Authors:** Guoliang Wang, Rui Fan, Ruirui Ji, Wenxin Zou, Daniel J. Penny, Nidhy P. Varghese, Yuxin Fan

**Affiliations:** John Welsh Cardiovascular Diagnostic Laboratory, Section of Cardiology, Department of Pediatrics, Texas Children’s Hospital, Baylor College of Medicine, 1102 Bates Ave, Suite 430.09, Houston, TX 77030 USA; Section of Pulmonology, Department of Pediatrics, Baylor College of Medicine, Houston, TX 77030 USA; Department of Pediatrics, Xijing Hospital, The Fourth Military Medical University, Xi’an, 710032 Shanxi China

**Keywords:** Pulmonary arterial hypertension, BMP9, Nonsense mutation

## Abstract

**Background:**

Pulmonary arterial hypertension (PAH) is a rare, progressive, fatal vascular disorder. Genetic predisposition plays vital roles in the development of PAH, with most mutations being identified in genes involved in the transforming growth factor beta (TGF-β) signaling pathways. Defects in the BMP9 gene have been documented in hereditary hemorrhagic telangiectasia (HHT), the most common inherited vascular disorder, which is occasionally associated with PAH. Selective enhancement of endothelial BMPR2 with BMP9 reverses pulmonary arterial hypertension.

**Case presentation:**

We report the case of a 5-year-old Hispanic boy who was diagnosed with severe PAH and right heart failure at 3 years of age. During his stay in the pediatric intensive care unit, treatment was initiated with inhaled nitric oxide and intravenous epoprostenol; he subsequently was transitioned to treprostinil, sildenafil, and prophylactic enoxaparin. Now, two years later, the child is asymptomatic on sildenafil, bosentan, subcutaneous treprostinil, and warfarin. Genetic screening revealed a novel homozygous nonsense mutation in the BMP9 gene (c.76C > T; p.Gln26Ter). The child had no telangiectasias or arteriovenous malformations; family history also was negative. Subsequent parental testing showed both parents were heterozygous for the same mutation, indicating that the child inherited the BMP9 mutant allele from each parent.

**Conclusion:**

To our knowledge, this is the first report of a BMP9 mutation in a patient with PAH. The homozygous nonsense mutation may account for the early onset and severity of PAH in this patient and also fit the ‘two-hit’ model we proposed previously. The absence of clinical symptoms for PAH in the parents may be due to incomplete penetrance or various expressivities of the BMP9 mutations. Our study expands the spectrum of phenotypes related to BMP9 mutations.

**Electronic supplementary material:**

The online version of this article (doi:10.1186/s12890-016-0183-7) contains supplementary material, which is available to authorized users.

## Background

Pulmonary arterial hypertension (PAH) is a progressive disease characterized by elevated mean pulmonary artery pressure (mPAP) of 25 mm Hg or more and pulmonary capillary wedge pressure (PCWP) of 15 mm Hg or less. In the current guideline, pulmonary vascular resistance (PVR) greater than 3 wood units was added as part of the hemodynamic definition of PAH [1]. The etiology of PAH is heterogeneous and incompletely understood. Gene mutations that have been described as promoters of PAH include mutations in the gene coding BMPR2 (bone morphogenetic protein receptor type II), a member of the transforming growth factor beta (TGF-β) signaling pathway, which has been identified in approximately 70 % of familial and up to 25 % of sporadic cases [2]. Other TGF-β signaling pathway partners, such as ACVRL1 (ALK1:activin receptor-like kinase type 1), ENG (endoglin), SMAD8, and SMAD4, are also reported to be involved in the pathogenesis of PAH [3]. Besides, potassium channel genes KCNA5 and KCNK3, NOTCH1, NOTCH3, TOPBP1 and EIF2AK4 (eukaryotic translation initiation factor 2) were documented as PAH disease causing genes [3–5]. BMP9/GDF2 may also be responsible for PAH by specifically activating the ALK1/BMPR2/ENG pathway and stimulating ET-1 (endothelin 1) via several SMAD pathways [6]. This present study is the first to identify a homozygous nonsense mutation in BMP9 in a patient with PAH and expands the spectrum of phenotypes related to BMP9 mutations.

## Case presentation

The proband, a 5-year-old boy, and his family members were enrolled in the study after informed written consent was obtained and this study was approved by the institutional review board. This former full-term child presented at 3 years of age with worsening dyspnea and exercise intolerance. He was noted to have perioral cyanosis, a new murmur, and cardiomegaly on chest X-ray. Echocardiogram showed a severely dilated main pulmonary artery and branch pulmonary arteries and dilated right atrium and right ventricle without any evidence of congenital heart disease. The predicted right ventricular systolic pressure was ~104 mmHg + right atrial pressure (simultaneous systemic blood pressure 102/78 mmHg), and the predicted pulmonary artery end-diastolic pressure was ~44 mmHg + RVEDP (right ventricular end-diastolic pressure). Brain-type natriuretic peptide (BNP) at admission was 4241 pg/mL (reference range, 0–100 pg/mL). He was diagnosed with PAH and started on subcutaneous infusion of 2 ng/kg/min of epoprostenol that was titrated upwards to a goal of 40 ng/kg/min, then transitioned to treprostinil of 80 ng/kg/min. During the admission, he was also started on sildenafil (1 mg/kg/dose) and prophylactic enoxaparin. Echocardiogram at discharge showed a right ventricular systolic pressure of ~59 mmHg (systemic blood pressure 90/50 mmHg). The right ventricle remained severely dilated with qualitatively moderate to severely depressed right ventricular systolic function. No pericardial effusion was noted. BNP was 650 pg/mL. Cardiac catheterization was done three months later on dual therapy and showed mild-moderate PH (Table 1). The cardiac catheterization data revealed that at rest, he had normal saturations and therefore there was no evidence of shunting or a mixing lesion. The blood was also oxygenated appropriately as it passed through the lungs. Mean artery pressures were elevated (>25 mmHg), capillary wedge pressure was normal (< 15 mmHg) and vascular resistance was elevated (> 2 WU/m2), consistent with diagnosis of PAH.

**Table 1:** Cardiac catheterization data

Saturations (%)					
	RA	RPA		LFA	Sys
REST	80	81		100	
On O_2_ (80 %)		91			100 %
On NO		89		100	
Pressures (mm Hg)					
	RA	RV	RPA	RPCW	LFA
REST	6/6/6	38/2/7	42/20/30	7/7/7	88/46/61
Isoproterenol 2mcg			60/25/43	9/9/8	101/45/64
On O_2_ (80 %)	6/5/5		42/20/29	7/7/7	89/42/60
On NO	7/6/6		43/18/29	7/7/7	85/41
Thermodilution					
	CO (ml/min)			CI (ml/min/m^2^)	
REST	3.2			4.55	
O_2_	3.1			4.43	
NO	3.3			4.07	

Using the above values including an assumed VO2 of 155 ml/min/m2 and Hgb 11.4 g/dL, calculations were the following: Qp = 5.2 L/min/m2, Qs = 5.2 L/min/m2; Qp:Qs = 1:1, PVR 4.4 WU/m2. No significant changes noted in pressures while on O2, NO or during administration of isoproterenol

*Abbreviations*: *RPA* right pulmonary artery, *LFA* left femoral artery, *S/D/M* systolic, diastolic, mean artery pressures, *RA* right atrium, *RV* right ventricle, *RPCW* right pulmonary capillary wedge, *CO* cardiac output, *CI* cardiac index, *Qp* pulmonary blood flow, *Qs* systemic blood flow, *PVR* pulmonary vascular resistance

Two years after his initial diagnosis, the child was doing well and was New York Heart Association functional class I. He is maintained on sildenafil (20 mg three times daily), bosentan (62.5 mg twice daily), treprostinil (121 ng/kg/min subcutaneously), and warfarin (3 mg daily). Echocardiogram showed trace tricuspid and pulmonary regurgitation and a mildly dilated main pulmonary artery. The right ventricle was moderately dilated with normal function. The family history is otherwise negative. No signs or symptoms of PAH or hereditary hemorrhagic telangiectasia (HHT) showed in either parent. They are from the same town in Mexico and denied any known consanguinity. Grandparents did not have HHT, nor did they have prolonged bleeding or PAH. Paternal grandmother was alive at time of case report; remaining grandparents were already deceased.

**Fig. 1:**
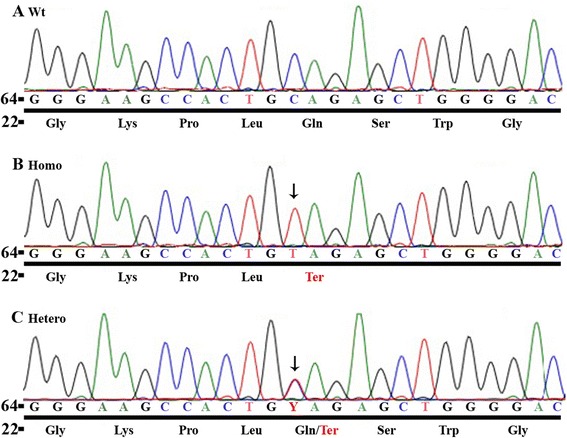
Genetic screening of the PAH-causing genes in the index patient and the parent. Panel (**a)** shows normal nucleotide fragment of BMP9 with corresponding amino acids underneath. Panel (**b**) identifies the homozygous nonsense mutation at nucleotide position 76 (c.76C > T) in the exon 1 of the BMP9 gene (NM_0016204.1), which causes the amino acid change from glutamine to tryptophan at peptide position of 26 (p.Gln26Ter) in the index patient. Panel (**c**) shows the heterozygous nonsense mutation at nucleotide position 76 (c.76C > T) in the exon 1 of the BMP9 gene (NM_0016204.1) found in the parent

For genetic analysis, DNA extraction, polymerase chain reaction (PCR) and sequence analysis were performed according to our protocols [7]. Genomic primer pairs were designed to amplify all of the coding regions and the intron-exon boundaries of known PAH-associated genes including BMPR2 [NM_001204.6], ACVRL1 [NM_001077401.1], ENG [NM_001114753.2], SMAD8 [NM_005905.5], SMAD4 [NM_005359.5], CAV1 [NM_001753.4], BMP9/GDF2 [NM_0016204.1], KCNK3 [NM_002246.2], KCNA5 [NM_002234.3], EIF2AK4 [NM_001013703.3], TOPBP1 [NM_007027.3], NOTCH1 [NM_017617.3], and NOTCH3 [NM_000435.2]. We identified a homozygous nonsense mutation (c.76C > T) of the BMP9/GDF2 gene (Fig. 1). This is a novel mutation that causes change from glutamine to terminator at amino acid position 26 (p.Gln26Ter). The parents are both heterozygotes for the same mutation, indicating that the child inherited a copy from each parent. Besides the mutation, we also found a number of polymorphisms (Additional file 1: Table S1).

## Conclusions

Various factors have been documented in the pathogenesis of PAH, and genetic mutations of genes involved in the TGF-β signaling pathway play essential roles in it. Herein we report a novel homozygous nonsense mutation in BMP9, another member of the TGF-β signaling pathway, in a patient with PAH.

As a member of the bone morphogenetic proteins (BMPs), BMP9 has been identified to be actively involved in the TGF-β signaling pathway by specifically binding to ALK-1, BMPR2, and ENG, which are causally related to PAH and HHT. The endothelial ALK1/BMPR2 pathway is constitutively activated by circulating BMP9, and BMP9 induces BMPR2 expression in endothelial cells in an ALK1-dependent manner [8]. In HHT pathogenesis, missense mutations within the orphan domain of ENG disrupt the high affinity interaction between ENG and BMP-9 [9]. BMP9-specific mutations were identified in patients with HHT [10], which is associated with a precapillary pattern of pulmonary hypertension that is histologically indistinguishable from idiopathic PAH. Moreover, a recent study showed that selective enhancement of endothelial BMPR2 with BMP9 reverses PAH [11]. These data clearly indicate that the BMP-9/ALK-1/BMPR2/ENG pathway is critical in the vascular pathogenesis of PAH/HHT. Disruption of BMP9/ALK-1/BMPR2/ENG pathway may, therefore, be important in the development of PAH/HHT-related vascular changes. Also, various studies have shown that BMP9 stimulates release of ET-1 in vascular endothelial cells via several SMAD pathways to regulate endothelial cell migration and angiogenesis [12].

In our patient, the homozygous nonsense mutation resulted in a premature truncation (c.76C > T, p.Gln26Ter). This truncated BMP9 may cause abnormal remodeling of pulmonary arterioles, which resulted in PAH due to the loss of function of BMP9. Loss of function of BMP9 may reduce the expression of BMPR2 and ALK-1 and disrupt its interaction with ENG, both of which may contribute to PAH pathogenesis. The homozygous nonsense mutation may account for the early onset and severity of PAH in this patient and also fit the ‘two-hit’ model we proposed [7]. The absence of clinical symptoms in the parents may be due to incomplete penetrance or various expressivities of the BMP9 mutations. We believe that our study has expanded the spectrum of phenotypes related to BMP9 mutations.

### Consent

Written informed consent was obtained from the patient and his parents for publication of this case report. A copy of the written consent is available for review by the Editor of this journal.

## Additional file

Additional file 1: Table S1. DNA alterations identified in the 13 PAH-associated genes. (DOC 40 kb)

## Abbreviations

BMPR2: bone morphogenetic protein receptor type II
HHT: hereditary hemorrhagic telangiectasia
mPAP: mean pulmonary artery pressure
PAH: pulmonary arterial hypertension
PCWP: pulmonary capillary wedge pressure
PVR: pulmonary vascular resistance
TGF-β: transforming growth factor beta
