# Supplementary material for: Novel homozygous BMP9 nonsense mutation causes pulmonary arterial hypertension: a case report
Source: BMC Pulm Med. 2016 Jan 22;16:17. doi: 10.1186/s12890-016-0183-7 (PMC4722683; doi:10.1186/s12890-016-0183-7)
Supplement: Additional file 1: Table S1. — DNA alterations identified in the 13 PAH-associated genes. (DOC 40 kb) [file 12890_2016_183_MOESM1_ESM.doc]

| **Patient** | **Genes** | **Nucleotide change** | **Amino acid change** | **Classification** |
| --- | --- | --- | --- | --- |
| **Index** | BMPR2 | - | - |  |
|  | ACVRL1 | - | - |  |
|  | CAV1 | - | - |  |
|  | ENG | - | - |  |
|  | SMAD4 | c.249+24A>G (hetero) | - |  |
|  | SMAD8 | - | - |  |
|  | **BMP9/GDF2** | **c.76C>T (homo)** | **p.Gln26Ter** | **Mutation** |
|  | KCNK3 | c.1185+13G>A (hetero) | - | Polymorphism |
|  | KCNA5 | c.381C>T (hetero) | p.Ser127Ser | Polymorphism |
|  |  | c.1129T>C (hetero) | p.Gly383Gly | Polymorphism |
|  | NOTCH1 | c.312T>C (hetero) | p.Asn104Asn | Polymorphism |
|  | NOTCH3 | c.4552C>A (hetero) | p.Leu1518Met | Polymorphism |
|  | TOPBP1 | c.1911T>C (hetero) | p.Val637Val | Polymorphism |
|  | EIF2AK4 | - | - |  |
| **The mother** | **BMP9/GDF2** | **c.76C>T (hetero)** | **p.Gln26Ter** | **Mutation** |
| **The father** | **BMP9/GDF2** | **c.76C>T (hetero)** | **p.Gln26Ter** | **Mutation** |

**Additional file 1: Table S1. DNA alterations identified in the 13 PAH-associated genes**
